# Ten‐year course of treated bipolar I disorder: The role of polarity at onset

**DOI:** 10.1002/brb3.2279

**Published:** 2021-10-09

**Authors:** María Yoldi‐Negrete, Ana Fresán‐Orellana, Mariana Jiménez‐Tirado, Sara Martínez‐Camarillo, Lino Palacios‐Cruz, Eduard Vieta, Hiram Ortega‐Ortiz, Claudia Becerra‐Palars, Doris Gutiérrez‐Mora, Beatriz Camarena Medellín

**Affiliations:** ^1^ Laboratorio de Epidemiología Clínica Subdirección de Investigaciones Clínicas Instituto Nacional de Psiquiatría Ramón de la Fuente Muñíz Ciudad de México México; ^2^ Enseñanza Hospital Médica Sur Ciudad de México México; ^3^ Subdirección de Servicios de Salud Petróleos Mexicanos Ciudad de México México; ^4^ Hospital Clínic Insitute of Neuroscience University of Barcelona IDIBAPS, CIBERSAM Catalonia Barcelona Spain; ^5^ Dirección de Servicios Clínicos Instituto Nacional de Psiquiatría Ramón de la Fuente Muñíz Ciudad de México México; ^6^ Departamento de Farmacogenética Subdirección de Investigaciones Clínicas Instituto Nacional de Psiquiatría Ramón de la Fuente Muñíz Ciudad de México México

**Keywords:** psychiatric disorders, psychiatry, psychosis

## Abstract

**Introduction:**

Early‐stage predictors of illness course are needed in bipolar disorder (BD). Differences among patients with a first depressive versus maniac/hypomanic episode have been stated, although in most studies, memory bias and time from onset to start of specialized treatment might interfere. The aim was to compare the first 10 years of illness course according to polarity at onset.

**Methods:**

49 type I BD patients admitted for treatment for a first‐time affective episode and a following 10‐year attendance to the institution were included. A retrospective year by year comparison according to polarity at onset (depressive (DPO) or maniac (MPO)) was performed. Cramer's *V* and Cohen *d* were computed to determine effect size.

**Results:**

59.2% (*n* = 29) started with MPO. Both groups were similar in demographic and social outcome characteristics, clinical features, and treatment variables. Patients with DPO reported more depressive episodes than MPO patients (*U* = 149.0 *p* < .001, Cohen's *d* = 0.87); both groups had a similar number of manic episodes. Only during the first year of follow‐up, suicide attempts (SA) were more frequent in patients with DPO while the presence of a psychotic episode and psychiatric hospitalizations were more frequent in the MPO group.

**Conclusion:**

According to these findings, it can be concluded that illness onset is only indicative of depressive predominant polarity but is not related to other poor prognostic variables after the first year of illness onset, in treated BD. SA in the first year of an affective disorder could represent a marker of BD.

## 1 INTRODUCTION

Bipolar disorder (BD) is a chronic and debilitating mental illness, present in approximately 2.4% of the world population (Merikangas et al., 2011). BD represents the sixth cause of disability worldwide and conveys a poor prognosis due to functional impairment and the presence of residual symptoms (Judd, 2002; Tohen et al., 2003).

Polarity at illness onset is defined as the pole (depressive or manic/hypomanic) at which a bipolar patient presents his or her first affective episode. Due to the high clinical heterogeneity of BD and its poor prognosis, polarity at illness onset has been studied as an early predictor of illness course in BD (Cremaschi et al., 2017; Daban et al., 2006; Etain et al., 2012; Forty et al., 2009; Perlis et al., 2005; Perugi et al., 2000; Tundo et al., 2015). The importance of studying polarity at illness onset is grounded on the possibility of a “glimpse” into the future course of illness, which may in turn guide clinicians in developing treatment and secondary prevention strategies at early stages of disease.

Polarity at illness onset was shown to be associated with predominant polarity, defined as the polarity of two thirds of total episodes (Carvalho et al., 2014; Daban et al., 2006; Perugi et al., 2000; Tundo et al., 2015). Patients with depressive first‐episode polarity (depressive polarity at onset, DPO) have shown a more chronic course of illness with greater number and longer duration of depressive episodes; whereas patients with maniac polarity at onset (MPO) tend to have more manic or hypomanic episodes (Daban et al., 2006; Etain et al., 2012; Forty et al., 2009; Perlis et al., 2005). The importance of the latter relies in the functional impairment that accompanies a greater number of episodes.

In terms of poor prognostic variables, DPO has been associated with more suicide attempts. On the other hand, MPO has been associated with a higher prevalence of psychotic symptoms and a greater number of hospitalizations (Cha et al., 2009; Chaudhury et al., 2007; Daban et al., 2006; Neves et al., 2009; Perlis et al., 2005; Perugi et al., 2000).

Previous studies have been of great value at identifying associations between polarity at illness onset and poor prognostic variables. Nonetheless, they lack control over important variables: reported polarity at onset was based on recollection from the patient; disease duration at study entry was different between studied patients; disease follow‐up also differed from one patient to another; report of number, polarity and hospitalizations of subsequent episodes was mostly obtained through interviews; patients were not treatment naïve at study entry (Table 1). The importance of controlling these variables rests on the fact that the number of episodes and poor prognostic variables may be influenced by the duration and management of disease and not by the polarity of onset itself. Also, data collection from clinical interviews, instead of the objective procurement of information from clinical records, entrails recall memory bias regarding the number and severity of episodes (Martino et al., 2016).

**Table 1:** Previous studies evaluating the course of illness according to first episode polarity

Author and year of publication	Sample size	Diagnosis	Years of illness evolution at study entry	Study design	Follow‐up duration	Method of evaluation	Main results
Perugi et al., 2000	320	BD I	DPO: 13.8 ± 10.3 MPO: 10.1 ± 9.4	Retrospective	Different for each individual	Structured interview and clinical records assessment	DPO: rapid cycling, more suicide attempts, greater number of episodes, greater prescription of antidepressants. MPO: more psychotic symptoms
Perlis et al., 2005	740	BD I	DPO: 25.3 ± 13.1 MPO: 22.7 ± 12.2	Retrospective	Different for each individual	Semi‐structured interview	DPO: females and earlier onset of disease, greater number of depressive episodes and incidence of anxiety disorders
Daban et al., 2006	300	BD I & II	DPO: 16.15 ± 11.4 MPO: 12.7 ± 10.8	Prospective	10 years	Structured interviews and clinical records assessment	DPO: longer duration of disease and greater number of episodes (total and depressive), more suicide attempts, later disease onset MPO: greater number of maniac episodes, more likely to develop psychotic symptoms and have hospitalizations
Kassem et al., 2006	971	BD I	Not specified	Retrospective	Different for each individual	Structured interviews and clinical records assessment	DPO: greater number of depressive episodes, panic attacks and alcohol dependence MPO: later disease onset
Chaudhury et al., 2007	113	BD I & II	DPO: 18.7 ± 12.2 MPO: 8 ± 9.2	Retrospective	Different for each individual	Semi‐structured interview	DPO: Greater number of depressive and total episodes, eightfold odd of suicide attempts MPO: greater incidence of psychotic symptoms and alcoholism
García López et al., 2009	296	BD I & II	Not specified	Prospective	1–4 years, assessment every 3 months	Clinical interview	DPO: more frequent in BD II MPO: more frequent in men
Cha et al., 2009	258	BD I	DPO: 7.7 ± 6.9 MPO: 7.9 ± 8.7	Retrospective	Different for each individual	Clinical records assessment	DPO: longer time lapse to confirmed diagnosis, greater number of suicide attempts MPO: greater number of hospitalizations
Forty et al., 2009	553	BD I	DPO: 22 ± 18 MPO: 15 ± 16	Retrospective	Different for each individual	Structured interviews and clinical records assessment	DPO: females, earlier age of onset, greater number of depressive episodes, more suicide attempts MPO: more psychotic symptoms
Neves et al., 2009	168	BD I & II	DPO: 17.5 ± 12.2 MPO:17.6 ± 12.5	Retrospective	Different for each individual	Semi‐structured interviews and clinical records assessment	DPO: females, greater number of suicide attempts MPO: more violent suicide attempts
Azorin et al., 2011	1089	BD I	DPO: 16.71 ± 11.9MPO:12.95 ± 11.2	Retrospective	Different for each individual.	Semi‐structured interviews and clinical records assessment	DPO: depressive temperaments, first episode triggered by stress and alcohol, more episodes, rapid cycling, suicide attempts, anxious comorbidity MPO: Hyperthymic temperamental predisposition, first episode triggered by substance abuse, psychotic features
Etain et al., 2012	1194	BD I	Not specified	Retrospective	Different for each individual	Clinical interview and database search	DPO: earlier disease onset, greater number of depressive episodes, suicidal attempts and alcohol misuse. MPO: greater number of maniac episodes and hospitalizations
Baldessarini et al. 2014	1081	BD I & II	15.7±12	Prospective	15 years, assessment every 1–3 months	Clinical records and life chart review	DPO: rapid cycling, more suicide attempts, more use of antidepressants MPO: greater probability of being unmarried and less education years, greater substance abuse, more hospitalizations
Tundo et al., 2015	407	BD I & II	DPO:13.5 ± 11.24 MPO:9.48 ± 8.81	Retrospective	Different for each individual	Semi‐structured interviews and clinical records assessment	DPO: more frequently diagnosed with BD II, lower rates of psychotic symptoms MPO: family history of psychosis and lower rates of suicide attempts
Cremaschi et al., 2017	362	BD I & II	DPO: 21 ± 12.98 MPO:15.8 ± 11.48	Retrospective	Different for each individual	Structured interviews and clinical records assessment	DPO: more suicide attempts, more frequently diagnosed with BD II, higher rate of lifetime stressful events, longer duration of most recent episode, higher use of antidepressants MPO: higher rate of psychosis and hospitalizations

Given these limitations in our current knowledge on the possible predictive capability of polarity at onset, we aimed to compare long‐term clinical variables in BD according to illness polarity in the first episode of disease (manic or depressive), in patients with an institutional follow‐up of their first 10 years of illness. We chose to assess variables from three major areas: (1) current social outcomes such as employment, years of education and marital status; (2) clinical outcomes in each year of follow‐up: presence and number of manic, depressive and mixed episodes; presence of psychotic episodes and suicide attempts and presence and number of psychiatric hospitalizations during illness course and; (3) the total number of psychiatric consultations received each year, assessed as a variable of service outcome.

## 2 METHOD

This study was approved by the Ethics Committee of the National Institute of Psychiatry at Mexico City with number CEI/C/018/2016. Participants gave their written consent for inclusion.

### 2.1 Subjects

Patients were identified from the Affective Disorders’ Clinic at the National Institute of Psychiatry *Ramón de la Fuente Muñíz* (INPRFM) in Mexico City, a highly specialized psychiatric facility dedicated to research, training, and inpatient and outpatient treatment for psychiatric conditions. The Affective Disorders’ Clinic is composed of treating psychiatrists specialized in bipolar disorder who follow international guidelines for the treatment of BD (Bandelow et al., 2012; Yatham et al., 2018) with an individualized evidence‐based treatment.

The present study included patients that had been admitted to the National Institute of Psychiatry for a first‐time mood episode, defined as mood symptoms sufficient to fulfill DSM criteria for either a depressive, a hypomanic or a manic episode (onset of first mood episode had occurred at most 2 years before admittance and had remained untreated during that period) and had from then‐on continued to attend this institution for at least 10 years, at a rate of at least one consultation a year in those 10 years, so memory bias could be significantly reduced (Martino et al., 2016). Additionally, patients who had received an initial diagnosis different from BD, during follow‐up were changed to bipolar disorder I, and from then‐on this remained the principal diagnosis according to clinical records. Only the first 10 years of institutional attendance were analyzed. Clinical records from a total of 116 cases with a first mood episode and current BD I diagnosis were reviewed. Sixty‐seven cases failed to have at least one consultation a year during the 10‐year period and were excluded from the study, therefore, 49 patients were included in the analysis.

### 2.2 Retrospective measurement

Included patients were admitted to the institution between 1991 and 2009. Diagnosis of bipolar disorder was established in the Affective Disorders’ Clinic by a face‐to‐face clinical interview of the patient and relatives with a psychiatrist specialized in affective disorders. Diagnosis was based on diagnostic criteria of the Diagnostic and Statistical Manual of Mental Disorders (DSM‐IIIR; DSM‐IV or DSM‐IV‐TR depending on the year of admission) and was confirmed by consensus of the psychiatrist who performed the interview and a senior psychiatrist of the clinic. Additionally, all included patients had diagnostic stability for the remaining years (despite changes in treating psychiatrist, BD remained the principal diagnosis).

The following data from each individual's first 10 years of institutional attendance was gathered from medical records:

‐ Current demographics (age, gender) and social outcomes (employment, years of education and marital status) were taken from the last visit of the assessment period (last visit in year number 10). Pharmacological treatment was as well recorded from this last visit, and not on a year‐by‐year basis.
‐ Clinical outcomes such as the presence and number of manic, depressive, and mixed episodes, presence of psychotic episodes and suicide attempts, presence and number of psychiatric hospitalizations and the total number of psychiatric consultations were gathered for each year of the follow‐up period. As the interest was on episode polarity, hypomanic and manic episodes were accounted for as manic episodes.

To ascertain reliability of variables extracted from clinical records, interrater reliability was performed, as described in previous studies of our work group (Yoldi‐Negrete et al., 2019).

### 2.3 Statistical analysis

All data were analyzed using SPSS version 21. Data is presented in frequencies and percentages for categorical variables and means and standard deviations (SD) for continuous variables. The comparison for each year of follow‐up according to patients’ initial illness polarity (depressive or maniac) was performed with Chi square tests (*χ* ^2^) or Mann–Whitney U tests (non‐normal distributed variables according to *p* < .05 in the Kolmogorov–Smirnov tests). Significance level for tests was established at *p* ≤ .05. Cramer's *V* and Cohen *d* were computed for the significant results obtained in the comparative analyses to determine their effect size. Values were interpreted as small (0.2–0.3), medium (0.4–0.7) and large (≥0.8).

## 3 RESULTS

### 3.1 Demographic characteristics, clinical features, and current social outcomes

Female patients accounted for 65.3% (*n* = 32) of the sample which had an average age of 37.5 years (S.D. = 9.5) and 13.4 (S.D. = 3.0) years of education at year number 10. Just over half of the patients were single and employed (51.0%, *n* = 25 each).

Just over half of the patients started treatment at the INPRFM in the same year as their illness onset (51.0%, *n* = 25), 28.6% (*n* = 14) the following year and 20.4% (*n* = 10) 2 years later. Age of first mood episode was reported at 27.7 years (S.D. = 8.7). Only 8.2% (*n* = 4) of the sample was stated to have a first‐degree family history of BD on clinical records. In year number 10, 57.1% (*n* = 28) were treated with magnesium valproate, 44.9% (*n* = 22) with lithium, 69.4% (*n* = 34) with other anticonvulsant medication and 65.3% (*n* = 32) were under antipsychotic treatment. Just over half of the patients (59.2%, *n* = 29) started with either a hypomanic or a manic episode, defined as manic polarity at onset (MPO group) and the remaining 40.8% (*n* = 20) had a depressive episode at onset, defined as DPO (group).

Both groups, MPO and DPO, were similar in demographic and social outcome characteristics, clinical features and treatment variables as shown in Table 2. Rapid cycling was not included in comparisons as only two patients presented with rapid cycling.

**Table 2:** Demographic, clinical and social outcome characteristics between patients with maniac (MPO) and depressive polarity at onset (DPO)

	Total sample *n* = 49	MPO *n* = 29	DPO *n* = 20	Statistics
*Demographic features*				
Gender—Female; *n* (%)	32 (65.3)	17 (58.6)	15 (75.0)	*χ* ^2^ = 1.4, *p* = .23
Age (years)*	37.5 9.5	37.8 10.7	37.2 7.8	*U* = 274.5, *p* = .75
*Social outcomes*				
Employment—Yes; *n (%)*	25 (51.0)	16 (55.2)	9 (45.0)	*χ* ^2^ = 0.4, *p* = .48
Marital status—Single; *n (%)*	25 (51.0)	15 (51.7)	10 (50.0)	*χ* ^2^ = 0.01, *p* = .90
Education (years)*	13.4 3.0	13.1 3.3	14.0 2.6	*U* = 255.5, *p* = .47
*Clinical features*				
Age of illness onset (years)*	27.7 8.7	28.2 9.0	27.1 8.4	*U* = 270.0, *p* = .68
*Current treatment—*Yes; *n* (%)				
Lithium	23 (46.9)	16 (55.2)	7 (35.0)	*χ* ^2^ = 1.9, *p* = .16
Magnesium valproate	30 (61.2)	17 (58.6)	13 (65.0)	*χ* ^2^ = 0.20, *p* = .65
Other anticonvulsants	34 (69.4)	18 (62.1)	16 (80.0)	*χ* ^2^ = 1.7, *p* = .18
Antipsychotic	33 (67.3)	21 (72.4)	12 (60.0)	*χ* ^2^ = 0.8, *p* = .36
BD family history—Yes; *n* (%)	4 (8.2)	2 (6.9)	2 (10.0)	*χ* ^2^ = 0.1, *p* = .69
*Psychiatric comorbidity*—Yes; *n(%)*				
Generalized anxiety disorder	10 (20.4)	4 (13.8)	6 (30.0)	*χ* ^2^ = 1.9, *p* = .16
Substance use disorder	10 (20.4)	6 (20.7)	4 (20.0)	*χ* ^2^ = 0.003, *p* = .95
Other psychiatric comorbidity	12 (24.5)	6 (20.7)	6 (30.0)	*χ* ^2^ = 0.5, *p *= .45

* Data reported in means and S.D.

**FIGURE 1:**
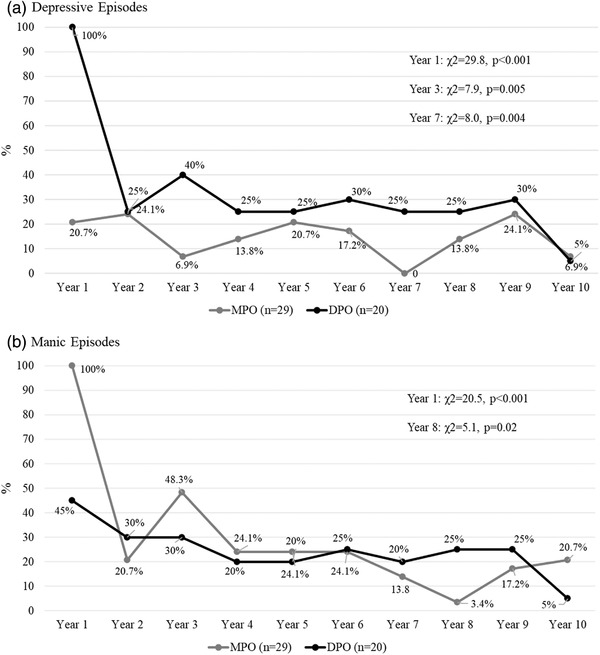
Percentage of patients presenting depressive (A) and manic (B) episodes during the 10‐year follow‐up

Figure 1a shows the proportion of patients who reported depressive episodes during the 10‐year institutional attendance. As can be seen, more patients with DPO reported depressive episodes during the institutional follow‐up period in the first (Cramer's *V* = 0.78), third (Cramer's *V* = 0.40) and seventh year (Cramer's *V* = 0.40). Number of depressive episodes was similar between groups (*p* > .05), mostly with 1 or 2 episodes and a minority, with 3 episodes (one patient of the DPO group in first year of follow‐up). During the first year, a higher proportion of patients with MPO exhibited manic episodes (Cramer's *V* = 0.64), with no differences between MPO or DPO in the remaining years, except for year 8, where more patients with DPO reported manic episodes (Cramer's *V* = 0.32) (Figure 1b). Comparable to what was observed with depressive episodes, the number of manic episodes in both groups ranged primarily between 1 and 2, and only in the first year, one patient of the DPO group reported three episodes. Less than 10% of patients in both groups reported mixed episodes during the 10‐year period, with up to two episodes each year, without differences between MPO and DPO groups. Considering the total number of episodes during the 10‐year period, patients with DPO reported more depressive episodes than MPO patients (mean 4.2, S.D. = 3.9 episodes vs. 1.6, S.D. = 1.5, *U* = 149.0 p < .001, Cohen's *d *= 0.87) while a similar number of manic episodes were observed in both groups (MPO mean = 3.2, S.D. = 2.0 vs. DPO mean 2.7, S.D. = 1.9, *U* = 240.0, *p* = .29).

**FIGURE 2:**
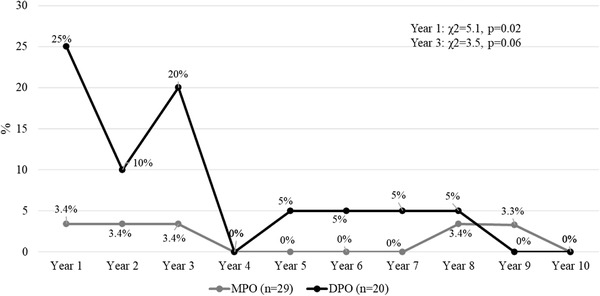
MPO and DPO patients with suicide attempts

**FIGURE 3:**
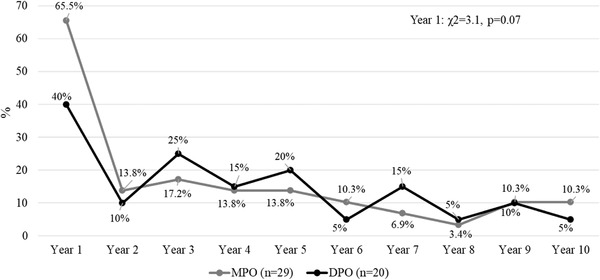
MPO and DPO patients with psychotic episodes

**FIGURE 4:**
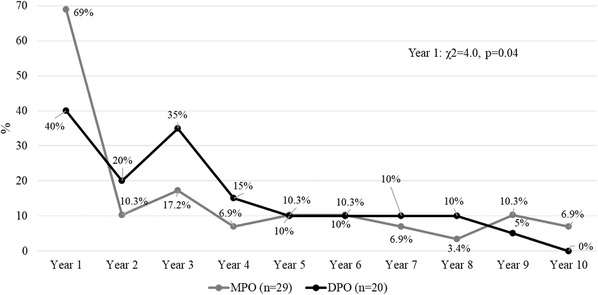
Hospitalized patients during the 10‐year follow‐up

The presence of a suicide attempt was more frequent in patients with DPO only during the first year (Cramer's *V* = 0.32) and a tendency to significant differences was observed in the third year, with similar percentages reported in both groups in the following years (Figure 2). The presence of a psychotic episode (Figure 3) was similar between groups during the 10‐year follow‐up (*p* > .05) while psychiatric hospitalizations (Figure 4) were more frequent in the MPO group during the first year of follow‐up (Cramer's *V* = 0.28), without differences between groups (*p* > .05) in the remaining years. The number of psychiatric hospitalizations in both groups ranged from 1 to 2 hospitalizations each year, without differences between groups during the 10‐year period considering the total number of hospitalizations (MPO mean = 2.0, S.D. = 1.7 vs. DPO = 1.8, S.D. = 1.1, *U* = 213.0, *p* = .88).

### 3.2 Service outcome: Number of psychiatric consultations during the 10‐year period

**FIGURE 5:**
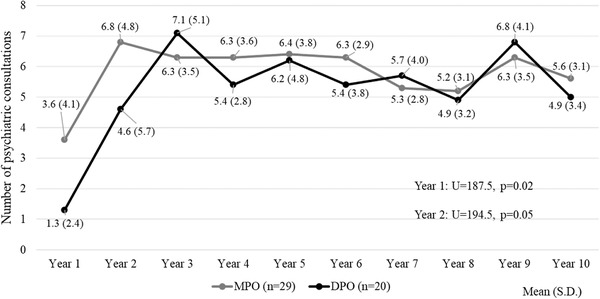
Number of total psychiatric consultations per‐year during the follow‐up

MPO patients had more psychiatric consultations than DPO patients during the first and second years (Cohen's *d *= 0.68 and 0.41, respectively), with a similar number of consultations in the remaining years (Figure 5) and also when considering the total number of psychiatric consultations during the 10‐year period (MPO mean total consultations = 58.4, S.D. = 19.2 vs. DPO mean total consultations = 52.4, S.D. = 22.6, *U* = 239.5, *p* = .30).

## 4 DISCUSSION

Search for prognostic factors as well as clues orienting to the best possible treatment early in the course of BD is still ongoing. Polarity at illness onset has been reported in several studies as one such factor. However, we believed avoiding memory bias in the recollection of the course of BD and having precise information on the first 10 years of illness evolution in every patient was necessary to establish the association between polarity at onset and illness course.

As found in previous studies, polarity at onset was associated with the polarity of subsequent depressive episodes (Carvalho et al., 2014; Colom et al., 2006; Perugi et al., 2000; Tundo et al., 2015): DPO patients had more depressive episodes than MPO patients, while there were no differences regarding the total number of manic episodes. This finding may serve as an indicator that DPO patients need more intensive prevention and management of depressive episodes to diminish the burden and impairment that come alongside. Indeed, an outstanding third of DPO patients had a suicide attempt in the first year of illness onset. The finding that a lifetime suicide attempt is higher in DPO patients than in MPO patients has been reported in several studies (Schaffer et al., 2015), and this study confirmed that the percentage of suicide attempts is higher year by year in the DPO group, although both groups have an important decrease after the third year. Although suicide attempts are complex phenomena, we believe that a suicide attempt in the first year of an affective disorder could be a marker of BD as a rate over 30% is much higher than the suicide‐attempt rate reported in the onset of other clinical entities: González‐Pinto et al. (2007) reported a suicide attempt in 8% of their sample at enrollment in their study of first‐episode affective and non‐affective psychotic inpatients; Shen et al. (2019) reported that 20.1% of their sample had a history of suicide attempts in their study of drug naïve patients with major depressive disorder, although these were not first‐episode patients. Furthermore, this rate is certainly much higher than that reported in general population (Centers for Disease Control and Prevention (CDC), 2005 ). However, conclusions on this matter will only be drawn with further studies using the proper methodology.

The difference in psychotic symptoms between groups was notable in the first year, with a much higher prevalence among MPO, present in over half the patients, but from that point on, differences tended to disappear. One should consider that it is very likely that only the most severe presentations of BD reach institutional management in their first year of illness evolution (Dagani et al., 2017) and this may account for such severe illness onset (suicide attempts in DPO and psychosis in MPO).

Hospital admittance is more prevalent in patients with a manic initial episode, despite the high prevalence of suicide attempts in the DPO group; we assume that this is due to the fact that manic episodes tend to be more disruptive and is congruent with other findings (Atigari et al., 2015), however, it could represent a red flag for treating physicians, as the severity of depression could be being underestimated.

After the first year, there were no differences in terms of poor prognostic variables such as number of hospitalizations and psychotic symptoms between groups. This finding is the opposite from what has been described by other studies (Azorin et al., 2011; Baldessarini et al., 2014; Cha et al., 2009; Chaudhury et al., 2007; Cremaschi et al., 2017; Daban et al., 2006; Etain et al., 2012; Forty et al., 2009; Garcia‐Lopez et al., 2009; Kassem et al., 2006; Neves et al., 2009; Perlis et al., 2005; Perugi et al., 2000; Tundo et al., 2015). We believe this could be due to differences in study methods, mainly the lack of memory bias in this study, but could also be attributed to illness course modification due to treatment: in this study, all patients were treatment naïve when institutional attendance started, but from then on, psychiatric treatment was ongoing. Treatment in psychiatry includes, but is not restricted to pharmacological therapy (Akiskal and Tohen (2011)), making change in disease course all the more probable. Indeed, patients with a BD diagnosis at our institution are motivated to assist to psychoeducational sessions which are routinely provided at our center (twice a year in group sessions). Individual and group psychotherapy is available and clinical sessions discussing difficult cases take place as frequently as needed by treating psychiatrists. A substantial reduction in the number of affective episodes, psychosis, suicide attempts and hospitalizations, is clear from the second year onward, and is mostly maintained over the 10‐year period.

There are several limitations in this study. Most limitations derive from the retrospective methodology used in the study: we had to exclude from analysis unreliable variables, many of which would have given a better understanding of the phenomenon (e.g., response to lithium; evolution of pharmacological treatment; certainty in the presence or absence of comorbid disorders; information regarding the patient's agreement for hospitalizations, among others). This was mainly due to substantial differences in clinical records as our study covers a time span of 30 years (the first 10 years of evolution for each patient, the first being admitted in 1991 and the last in 2009): policies, treating physicians, guidelines and available therapeutic options changed considerably during this time. However, many interesting variables were reliable, notably the number and polarity of episodes during these years, which we believe adds important information to our current knowledge on polarity in type I BD.

Also, it is difficult to generalize these findings as this sample centered on type I BD. The inclusion of patients with type II BD was originally intended. However, the sample reached for BD II was too small (*n* = 9) to allow for comparisons between BD I and BD II. We decided to sacrifice the representativeness in order to gain methodological strength.

The fact that this is a population of treated BD and one with excellent adherence, also affects generalizability. These characteristics make this population less vulnerable to severe clinical outcomes and relapses due to their strict medical supervision and probably due to patient's insight (de Barros Pellegrinelli et al., 2013). It is also very probable that these patients represent a population with a severe form of disease onset, and probably a very effective support network as all reached a highly specialized facility in the first year, which is very uncommon: a several years delay in specialized treatment for psychiatric disorders is sadly the rule rather than the exception, and seems to be a worldwide problematic (del Valle et al., 2017; Fikretoglu et al., 2010; Goldberg et al., 2019; Green et al., 2012; Ki et al., 2014; Stagnaro et al., 2019).

The small sample size must also be mentioned, although one must consider the difficulty in fulfilling inclusion criteria, having the prior statement in consideration.

Yet another limitation that must be stated is related to differences in diagnostic criteria between DSM‐IV and DSM‐5 (Kessing et al., 2021): as previous criteria of DSM were followed for the detection of polarity at onset, we cannot rule‐out the possibility that some patients might have had a first hypomanic episode of short duration as their first affective episode. Little is known on the subset of patients with short duration hypomanias (Miller et al., 2016), and the specific question on the evolution from a first affective episode of these characteristics must be addressed in future studies.

To our knowledge, this is the first study that evaluates the illness course of treated bipolar disorder year by year in the first 10 years of illness evolution and compares it according to polarity at illness onset. The main strength of the present study relies on the fact that included patients started the follow‐up at disease onset, therefore reducing the possibility of recall bias and the possible confounding effect of untreated BD. Another important aspect of the studied population is the control over disease duration since all patients were followed during their first 10 years of illness course (with at most 2 years variation).

In conclusion, our study shows that in treated BD, illness onset is only indicative of depressive predominant polarity but is not related to other poor prognostic variables; adds evidence of the effectiveness of psychiatric treatment in this disorder; and highlights suicide attempts in the first year of an affective disorder as a possible marker of BD. Further longitudinal studies in different populations are needed to allow for generalization of these findings as well as comparisons between suicide attempts in the first year of other affective disorders.

### DATA SHARING

The data that support the findings of this study are available from the corresponding author upon reasonable request.

### TRANSPARENT PEER REVIEW

The peer review history for this article is available at https://publons.com/publon/10.1002/brb3.2279

## References

[brb32279-bib-0001] Akiskal, H. S. , Tohen, M. (2011). Bipolar psychopharmacotherapy. John Wiley & Sons, Ltd. 10.1002/9780470975114

[brb32279-bib-0002] Atigari, O. V. , Harris, M. , Le Noury, J. , & Healy, D . (2015). Bipolar disorder and its outcomes: Two cohorts, 1875–1924 and 1994–2007, compared. History of Psychiatry, 27(1), 75–84. 10.1177/0957154X15624601 26769392

[brb32279-bib-0003] Azorin, J. M. , Kaladjian, A. , Adida, M. , Fakra, E. , Hantouche, E. , & Lancrenon, S . (2011). Correlates of first‐episode polarity in a French cohort of 1089 bipolar I disorder patients: Role of temperaments and triggering events. Journal of Affective Disorders, 129(1‐3), 39–46. 10.1016/j.jad.2010.08.020 20855116

[brb32279-bib-0004] Baldessarini, R. J. , Tondo, L. , & Visioli, C . (2014). First‐episode types in bipolar disorder: Predictive associations with later illness. Acta Psychiatrica Scandinavica, 129(5), 383–392. 10.1111/acps.12204 24152091

[brb32279-bib-0005] Bandelow, B. , Sher, L. , Bunevicius, R. , Hollander, E. , Kasper, S. , Zohar, J. , & Möller, H. J. (2012). WFSBP task force on mental disorders in primary care; WFSBP task force on anxiety disorders, OCD and PTSD. Guidelines for the pharmacological treatment of anxiety disorders, obsessive‐compulsive disorder and posttraumatic stress disorder in primary care. International Journal of Psychiatry in Clinical Practice, 16(2), 77–84. 10.3109/13651501.2012.667114 22540422

[brb32279-bib-0006] Carvalho, A. F. , McIntyre, R. S. , Dimelis, D. , Gonda, X. , Berk, M. , Nunes‐Neto, P. R. , Cha, D. S. , Hyphantis, T. N. , Angst, J. , & Fountoulakis, K. N. (2014). Predominant polarity as a course specifier for bipolar disorder: A systematic review. Journal of Affective Disorders, 163, 56–64. 10.1016/j.jad.2014.03.035 24836088

[brb32279-bib-0007] Centers for Disease Control and Prevention, National Centers for Injury Prevention and Control. Web‐based Injury Statistics Query and Reporting System (WISQARS) [online]. (2005) {cited 2021 Jul 14}. Available from: www.cdc.gov/injury/wisqars

[brb32279-bib-0008] Cha, B. , Kim, J. H. , Ha, T. H. , Chang, J. S. , & Ha, K . (2009). Polarity of the first episode and time to diagnosis of bipolar I disorder. Psychiatry Investigation, 6(2), 96–101. 10.4306/pi.2009.6.2.96 20046381 PMC2796048

[brb32279-bib-0009] Chaudhury, S. R. , Grunebaum, M. F. , Galfalvy, H. C. , Burke, A. K. , Sher, L. , Parsey, R. V. , Everett, B. , Mann, J. J. , & Oquendo, M. A . (2007). Does first episode polarity predict risk for suicide attempt in bipolar disorder? Journal of Affective Disorders, 104(1–3), 245–250. 10.1016/j.jad.2007.02.022 17434597 PMC2151386

[brb32279-bib-0010] Colom, F. , Vieta, E. , Daban, C. , Pacchiarotti, I. , & Sánchez‐Moreno, J . (2006). Clinical and therapeutic implications of predominant polarity in bipolar disorder. Journal of Affective Disorders, 93(1–3), 13–17. 10.1016/j.jad.2006.01.032 16650901

[brb32279-bib-0011] Cremaschi, L. , Dell'Osso, B. , Vismara, M. , Dobrea, C. , Buoli, M. , Ketter, T. A. , & Altamura, A. C. (2017). Onset polarity in bipolar disorder: A strong association between first depressive episode and suicide attempts. Journal of Affective Disorders, 209, 182–187. 10.1016/j.jad.2016.11.043 27936451

[brb32279-bib-0012] Daban, C. , Colom, F. , Sanchez‐Moreno, J. , García‐Amador, M. , & Vieta, E . (2006). Clinical correlates of first‐episode polarity in bipolar disorder. Comprehensive Psychiatry, 47(6), 433–437. 10.1016/j.comppsych.2006.03.009 17067865

[brb32279-bib-0013] Dagani, J. , Signorini, G. , Nielssen, O. , Bani, M. , Pastore, A. , Girolamo, G. , & Large, M . (2017). Meta‐analysis of the interval between the onset and management of bipolar disorder. Canadian Journal of Psychiatry Revue Canadienne De Psychiatrie, 62(4), 247–258. 10.1177/0706743716656607 27462036 PMC5407546

[brb32279-bib-0014] de Barros Pellegrinelli, K. , de O Costa, L. F. , Silval, K. I. , Dias, V. V. , Roso, M. C. , Bandeira, M. , Colom, F. , & Moreno, R. A . (2013). Efficacy of psychoeducation on symptomatic and functional recovery in bipolar disorder. Acta Psychiatrica Scandinavica, 127(2), 153–158. 10.1111/acps.12007 22943487

[brb32279-bib-0015] del Valle, G. , Belloch, A. , & Carrió, C . (2017). The long and complex road in the search for treatment for mental disorders: An analysis of the process in five groups of patients. Psychiatry Research, 253, 1–8. 10.1016/j.psychres.2017.03.024 28319785

[brb32279-bib-0016] Etain, B. , Lajnef, M. , Bellivier, F. , Mathieu, F. , Raust, A. , Cochet, B. , Gard, S. , M'Bailara, K. , Kahn, J. P. , Elgrabli, O. , Cohen, R. , Jamain, S. , Vieta, E. , Leboyer, M. , & Henry, C . (2012). Clinical expression of bipolar disorder type I as a function of age and polarity at onset. Journal of Clinical Psychiatry, 73(04), e561–e566. 10.4088/JCP.10m06504 22579163

[brb32279-bib-0017] Fikretoglu, D. , Liu, A. , Pedlar, D. , & Brunet, A . (2010). Patterns and predictors of treatment delay for mental disorders in a nationally representative, active Canadian military sample. Medical Care, 48(1), 10–17. 10.1097/MLR.0b013e3181bd4bf9 19956080

[brb32279-bib-0018] Forty, L. , Jones, L. , Jones, I. , Smith, D. J. , Caesar, S. , Fraser, C. , Gordon‐Smith, K. , Hyde, S. , & Craddock, N . (2009). Polarity at illness onset in bipolar I disorder and clinical course of illness. Bipolar Disorders, 11(1), 82–88. 10.1111/j.1399-5618.2008.00654.x 19133970

[brb32279-bib-0019] Garcia‐Lopez, A. , De Dios‐Perrino, C. , & Ezquiaga, E . (2009). Polarity of the first episode and predominant polarity in a cohort of bipolar outpatients. European Neuropsychopharmacology, 19, S571. 10.1016/S0924-977X(09)70912-5

[brb32279-bib-0020] Goldberg, S. B. , Simpson, T. L. , Lehavot, K. , Katon, J. G. , Chen, J. A. , Glass, J. E. , Schnurr, P. P. , Sayer, N. A. , & Fortney, J. C . (2019). Mental health treatment delay: A comparison among civilians and veterans of different service eras. Psychiatric Services, 70(5), 358–366. 10.1176/appi.ps.201800444 30841842 PMC6510540

[brb32279-bib-0021] González‐Pinto, A. , Aldama, A. , González, C. , Mosquera, F. , Arrasate, M. , & Vieta, E . (2007). Predictors of suicide in first‐episode affective and nonaffective psychotic inpatients: Five‐year follow‐up of patients from a catchment area in Vitoria, Spain. Journal of Clinical Psychiatry, 68(2), 242–247. 10.4088/JCP.v68n0209 17335322

[brb32279-bib-0022] Green, A. C. , Hunt, C. , & Stain, H. J . (2012). The delay between symptom onset and seeking professional treatment for anxiety and depressive disorders in a rural Australian sample. Social Psychiatry and Psychiatric Epidemiology, 47(9), 1475–1487. 10.1007/s00127-011-0453-x 22116199

[brb32279-bib-0023] Judd, L. L . (2002). The long‐term natural history of the weekly symptomatic status of bipolar I disorder. Archives of General Psychiatry, 59(6), 530–537. 10.1001/archpsyc.59.6.530 12044195

[brb32279-bib-0024] Kassem, L. , Lopez, V. , Hedeker, D. , Steele, J. , Zandi, P. , & McMahon, F . (2006). Familiality of polarity at illness onset in Bipolar affective Disorder. American Journal of Psychiatry, 163, 1754–1759. 10.1176/ajp.2006.163.10.1754 17012686

[brb32279-bib-0025] Kessing, L. V. , González‐Pinto, A. , Fagiolini, A. , Bechdolf, A. , Reif, A. , Yildiz, A. , Etain, B. , Henry, C. , Severus, E. , Reininghaus, E. Z. , Morken, G. , Goodwin, G. M. , Scott, J. , Geddes, J. R. , Rietschel, M. , Landén, M. , Manchia, M. , Bauer, M. , Martinez‐Cengotitabengoa, M. , Andreassen, O., A., Ritter, P., Kupka, R ., Licht, R. W., Nielsen, R. E., Schulze, T. G., Hajek, T., Lagerberg, T., V.,Bergink, V. & Vieta, E . (2021). DSM‐5 and ICD‐11 criteria for bipolar disorder: Implications for the prevalence of bipolar disorder and validity of the diagnosis – A narrative review from the ECNP bipolar disorders network. European Neuropsychopharmacology, 47, 54–61. 10.1016/j.euroneuro.2021.01.097 33541809

[brb32279-bib-0026] Ki, M. , Paik, J. W. , Choi, K. S. , Ryu, S. H. , Han, C. , Lee, K. , Ham, B. J. , Chang, H. S. , Won, E. S. , Jun, T. Y. , & Lee, M. S . (2014). Delays in depression treatment among Korean population. Asia‐Pacific Psychiatry, 6(4), 414–424. 10.1111/appy.12140 25103868

[brb32279-bib-0027] Martino, D. J. , Marengo, E. , Igoa, A. , Scápola, M. , Urtueta‐Baamonde, M. , & Strejilevich, S. A . (2016). Accuracy of the number of previous episodes reported by patients with bipolar disorder. Comprehensive Psychiatry, 65, 122–127. 10.1016/j.comppsych.2015.11.005 26774000

[brb32279-bib-0028] Merikangas, K. , Jin, R. , He, J. , Kessler, R. , & Al, E . (2011). Prevalence and correlates of bipolar spectrum disorder in the world mental health survey initiative. Archives of General Psychiatry, 68(3), 241–251. 10.1001/archgenpsychiatry.2011.12 21383262 PMC3486639

[brb32279-bib-0029] Miller, S. , Dennehy, E. B. , & Suppes, T . (2016). The prevalence and diagnostic validity of short‐duration hypomanic episodes and major depressive episodes. Current Psychiatry Reports, 18(3), 1–7. 10.1007/s11920-016-0669-2 26830885

[brb32279-bib-0030] Neves, F. S. , Malloy‐Diniz, L. F. , Barbosa, I. G. , Brasil, P. M. , & Corrêa, H . (2009). Bipolar disorder first episode and suicidal behavior: Are there differences according to type of suicide attempt? TT ‐ A polaridade do primeiro episódio no transtorno bipolar é um preditor para tentativa de suicídio (violenta e não violenta) futura? Brazilian Journal of Psychiatry, 31(2), 114–118. 10.1590/S1516-44462009000200006 19578682

[brb32279-bib-0031] Perlis, R. H. , Delbello, M. P. , Miyahara, S. , Wisniewski, S. R. , Sachs, G. S. , & Nierenberg, A. A . (2005). Revisiting depressive‐prone bipolar disorder: Polarity of initial mood episode and disease course among bipolar I systematic treatment enhancement program for bipolar disorder participants. Biological Psychiatry, 58(7), 549–553. 10.1016/j.biopsych.2005.07.029 16197928

[brb32279-bib-0032] Perugi, G. , Micheli, C. , Akiskal, H. S. , Madaro, D. , Socci, C. , Quilici, C. , & Musetti, L. (2000). Polarity of the first episode, clinical characteristics, and course of manic depressive illness: A systematic retrospective investigation of 320 bipolar I patients. Comprehensive Psychiatry, 41(1), 13–18. 10.1016/S0010-440X(00)90125-1 10646613

[brb32279-bib-0033] Schaffer, A. , Isometsä, E. T. , Tondo, L. , Moreno, D. H. , Turecki, G. , Reis, C. , Cassidy, F. , Sinyor, M. , Azorin, J. M. , Kessing, L. V. , Ha, K. , Goldstein, T. , Weizman, A. , Beautrais, A. , Chou, Y. H. , Diazgranados, N. , Levitt, A. J. , Zarate, C. A., Jr , Rihmer, Z. , & Yatham, L. N . (2015). International society for bipolar disorders task force on suicide: Meta‐analyses and meta‐regression of correlates of suicide attempts and suicide deaths in bipolar disorder. Bipolar Disorders, 17(1), 1–16. 10.1111/bdi.12271 PMC6296224 25329791

[brb32279-bib-0034] Shen, Y. , Wu, F. , Zhou, Y. , Ma, Y. , Huang, X. , Ning, Y. , Lang, X. , Luo, X. , & Zhang, X . (2019). Association of thyroid dysfunction with suicide attempts in first‐episode and drug naïve patients with major depressive disorder. Journal of Affective Disorders, 259, 180–185. 10.1016/j.jad.2019.08.067 31446378

[brb32279-bib-0035] Stagnaro, J. C. , Cia, A. H. , Vommaro, H. , Sustas, S. , Vázquez, N. , Serfaty, E. , Kessler, R. C. , & Benjet, C. (2019). Delays in making initial treatment contact after the first onset of mental health disorders in the Argentinean Study of Mental Health Epidemiology. Epidemiology and Psychiatric Sciences, 28(2), 240–250. 10.1017/S2045796018000094 29540248 PMC6998935

[brb32279-bib-0036] Tohen, M. , Zarate, C. A., Jr , Hennen, J. , Khalsa, H. M. , Strakowski, S. M. , Gebre‐Medhin, P. , Salvatore, P. , & Baldessarini, R. J . (2003). The McLean‐Harvard first‐episode mania study: Prediction of recovery and first recurrence. American Journal of Psychiatry, 160(12), 2099–2107. 10.1176/appi.ajp.160.12.2099 14638578

[brb32279-bib-0037] Tundo, A. , Musetti, L. , Benedetti, A. , Berti, B. , Massimetti, G. , & Dell'Osso, L . (2015). Onset polarity and illness course in bipolar I and II disorders: The predictive role of broadly defined mixed states. Comprehensive Psychiatry, 63, 15–21. 10.1016/j.comppsych.2015.07.018 26555487

[brb32279-bib-0038] Yatham, L. N. , Kennedy, S. H. , Parikh, S. V. , Schaffer, A. , Bond, D. J. , Frey, B. N. , Sharma, V. , Goldstein, B. I. , Rej, S. , Beaulieu, S. , Alda, M. , MacQueen, G. , Milev, R. V. , Ravindran, A. , O'Donovan, C. , McIntosh, D. , Lam, R. W. , Vazquez, G. , Kapczinski, F. , … & Berk, M . (2018). Canadian network for mood and anxiety treatments (CANMAT) and international society for Bipolar Disorders (ISBD) 2018 guidelines for the management of patients with bipolar disorder. Bipolar Disorders, 20(2), 97–170. 10.1111/bdi.12609 29536616 PMC5947163

[brb32279-bib-0039] Yoldi‐Negrete, M. , Morera, D. , Palacios‐Cruz, L. , Camarena, B. , Ortega, H. , Castañeda‐Franco, M. , Becerra‐Palars, C. , Martino, D. , Strejilevich, S. , & Fresan, A . (2019). Subsyndromal anxiety: Does it affect the quality of life? A study on euthymic patients with bipolar disorder. European Journal of Psychiatry, 33(4), 159–164. 10.1016/j.ejpsy.2019.06.005https

